# How to best assess shedder status: a comparison of popular shedder tests

**DOI:** 10.1007/s00414-024-03351-8

**Published:** 2024-11-07

**Authors:** Darya Ali, Roland A. H. van Oorschot, Adrian Linacre, Mariya Goray

**Affiliations:** 1 College of Medicine and Public Health, Flinders University, Bedford Park, South Australia Australia; 2 Office of the Chief Forensic Scientist, Victoria Police Forensic Services Department, Macleod, VIC Australia; 3 School of Agriculture, Biomedicine and Environment, La Trobe University, Bundoora, VIC Australia; 4 College of Science and Engineering, Flinders University, Bedford Park, South Australia Australia

**Keywords:** Touch DNA, Shedder status, Shedder test, Handprint, Handheld tube, Diamond dye

## Abstract

“Shedder status” describes the inherent variation between individuals to leave touch DNA on a surface through direct contact. Depending on the amount and quality of DNA or cellular deposition, individuals are typically deemed high, intermediate, or low shedders. Although many shedder tests have been described, variability in study design and categorisation criteria has limited the ability of researchers to accurately compare results, as well as accrue the necessary population data. As activity level reporting becomes more common, the need for reliable and standardised testing increases. To assess reproducibility, this study compared shedder status data generated by six participants using three different shedder tests, as modified from the literature. This involved DNA quantification and profiling of a handprint made on a glass plate, DNA quantification and profiling of a grip mark made on a plastic conical tube, and cell scoring of a Diamond™ Dye-stained fingermark. All participants washed and dried their hands fifteen minutes before each deposit. To assess the impact of behaviour on shedder designation, participants either refrained from activity or went about their daily tasks during this wait. The shedder status of participants changed between tests, as DNA-based testing often generated lower shedder statuses than cell scores. Further, when different categorisation methods were applied to a single test, intra-person variability increased as the number of shedder designations increased from two (low/high) to five (low/low-intermediate/intermediate/intermediate-high/high). Moving forward, the utilisation of a single shedder test and standardised categorisation criteria is needed to employ shedder testing in forensic casework.

**Supplementary Information:**

The online version contains supplementary material available at 10.1007/s00414-024-03351-8.

## Introduction

Since the first observation of varying touch DNA quantities deposited by different individuals [1] and the subsequent, inaugural shedder type assessment conducted by Lowe et al. [2], researchers have concluded that shedder status, or one’s propensity towards shedding DNA, has an irrefutable impact on touch DNA deposition and subsequent transfer [3–17]. As a result, the recovery of touch DNA from crime scenes has provided the criminal justice system with growing evidence regarding perpetrator activity, as forensic scientists are often asked to provide testimony regarding the presence and transfer of incriminatory DNA.

However, contextualising a criminal act is challenging, as recovered samples often generate mixed DNA profiles, representing contacts made by individuals during the commission of a crime, as well as by others, innocently, in the time preceding the event. If known, the shedder status of an individual may provide an inference of how much DNA is expected to be found on an item and what their relative contributions to a DNA mixture is likely to be [13, 18].

Shedder status is also relevant in the context of indirect DNA transfer, where transfer occurs through a third party or intermediary surface [14]. Considering the potential, grave implications of indirect transfer, Bayesian networks are now being used to address questions regarding activity level reporting, the results of which can be interpreted by forensic scientists and presented in court. These networks require input data, including amount of DNA available for transfer, a factor largely influenced by shedder status, and the alleged activities conducted [19]. As such, knowledge of a perpetrator’s shedder status may be invaluable in assessing their role in an alleged crime.

To properly assess an individual’s shedder status at the time of an alleged offence, multiple considerations and subsequent investments must be made, including: 1) studies to determine how shedder tests are best performed; 2) studies to establish the best methods for categorising individuals based on said shedder test results; 3) studies to determine the degree of and cause for variation in an individual’s shedder status over time; and 4) once a standardised, validated test and categorisation method is available, the development of appropriate procedures for determining who should be tested, when and how often, and by whom. In this study, we focus on the first two elements by assessing three commonly used shedder tests and their associated categorisation methodologies to see if their application provides consistent shedder outcomes for six individuals or if, instead, variation in outcomes suggest a need for further improvement to and standardisation of shedder testing and categorisation methodologies.

Although several studies have employed shedder testing of individuals (see Supplementary Data 1), there is no standardised approach to how these tests are conducted or how individual results are categorised. Inconsistencies in previously conducted shedder tests include: within the pre-deposit phase, activity restrictions, use of handwashing, and the time between handwashing and deposition [2, 5, 11, 20, 21]; within the deposition phase, contacted substrate [16, 22–24] and the manner and duration of contact [2, 6, 9, 11, 17, 24–27]; and within the post-deposition phase, the methodologies applied to collect, extract, quantitate, and profile recovered DNA, as well as the number of replicates conducted per person [2, 17, 20, 27–29]. In respect to shedder categorisation, there are inconsistencies regarding the parameters and number of classification categories used, as well as the nomenclature used to describe said categories (see Supplementary Data 1).

Early studies used a binary system of classification, recognizing “good” and “bad” shedders [2, 18, 21, 24, 30], while a ternary classification that recognizes high, intermediate, and low shedders is now widely used [11, 17, 27, 31]. Goray & van Oorschot expanded shedder classification to include five overlapping shedder status categories [15], while Petcharoen et al. recently concluded that shedder types are a continuum with no discrete boundaries [32].

High shedders typically describe individuals who are more likely to leave their own DNA on surfaces relative to their low shedder counterparts, consistently making them the sole or majority contributor in a mixed deposit [17]. Comparatively, low shedders have a predisposition towards transferring little self and high non-self-DNA to surfaces, resulting in minor or negligible contributions to a mixture [17]. Intermediate shedders, who have been found to make up the largest of the three sub-groups [17, 20, 27], vary in their relative shedder behaviour, as they may mimic the behaviours of a low or high shedder, producing inconsistent results [15, 33–35].

As such, shedder tests often describe an acceptable level of intra-personal variation, which is generally mitigated with multiple deposits [2, 17, 21, 27], as participants are thought to maintain their shedder status within an experimental design, even when tested on different days or years apart [15]. Individual differences in deposits are associated with changes in activity, behavioural, and environmental factors, including recent intimate contact [15, 17], oral intake [34], glove use [17, 18, 35], moisturizer application [17, 36], and untreated skin conditions [30]. Non-modifiable factors, including age [18, 27, 35, 37] and biological sex [11, 17, 18, 20, 27], have also been shown to impact shedder status.

To date, dozens of shedder tests have been described (see Supplementary Data 1), but few direct comparisons have been made to determine whether an individual maintains their status between different test types [11, 31, 34, 38]. Moreover, few considerations have been given to the impact of classification criteria on shedder status. Looking to the future, without general acceptance regarding what constitutes a shedder test and what classification criteria is to be applied, the role of shedder testing in forensic casework will remain largely immaterial.

This study aims to determine whether six individuals maintain their shedder status designation between three different shedder tests [11, 15, 18]. These tests were all heavily cited in the literature and capture the diversity in shedder status testing. Our primary purpose was not to select a “best” test or modify past studies in light of today’s recommended practices. Instead, we replicated studies as described in the literature, with modifications only being made to allow for an accurate comparison of tests completed on separate days. Such modifications included standardised handwashing, which was implemented in one of the three initial shedder tests [11] and used to minimise the impact of pre-deposit behaviour, as well as the use of a “clean” and a “dirty” hands condition to assess the impact of activity on shedder status. Method-specific categorisation criteria was then applied to all three tests, where relevant, to determine the impact of classification criteria on overall shedder categorisation.

## Materials and methods

### Experimental design

#### Participants

Six participants (referred to as Participants 1–6 hereafter) were recruited as donors for all three experiments. Two of the selected participants were a high (Participant 2) and low shedder (Participant 4), based on previous Diamond™ Dye (DD) testing (unpublished). Two of the participants were male (2, 6) and four were female with ages that ranged from 18 to 65-years-old. Of the six participants, half were right-handed (3, 4, 5) and half were left-handed (1, 2, 6). Additional participant factors (age, gender, living arrangements, known skin conditions, etc.) are described in Supplementary Data 2.

#### Handwashing and activity

To minimize the impact of pre-testing behaviour and activity, participants were instructed to wash their hands for 15–20 s with Softsoap^©^ Antibacterial liquid soap (Colgate-Palmolive Company, Manhattan, NY, USA) before drying their hands with paper towel, all while being observed. All sample deposits were made 15 min after handwashing.

Further, for this study, a “clean” and a “dirty” hands conditions were used to assess the impact of activity on shedder status. In the clean hands condition, participants were instructed to not touch anything for 15 min post-handwashing to assess the intrinsic amount of DNA or cells accumulated during that time period. To ensure compliance, participants were supervised during the wait period. In the dirty hands condition, participants were instructed to go back to their normal, daily activities with few exceptions. Participants were instructed not to eat or drink, actively touch other people, wear gloves, or re-wash or sanitize their hands. Participants were also kept from touching shared laboratory surfaces including door handles throughout the 15-min wait period. Surveys were collected at each deposit to ascertain activity information.

#### Replication

With the exception of Participant 1, all samples were collected over the same five-month period, with most participants depositing one sample per week. Participant 1 had a full set of samples (36 samples over Tests 1, 2, and 3) collected over the same five-month period, as well as an additional four samples (two clean and two dirty) collected on glass slides over a period of four weeks, approximately 12 months after they made their first deposits.

#### Selected shedder tests

The three shedder tests selected (DNA quantification and profiling of a handprint on a glass plate, DNA quantification and profiling of a grip mark on a plastic conical tube, and cell scoring of a Diamond™ Dye-stained fingermark) were replicated from the literature with slight modifications [11, 15, 18] (see Supplementary Data 3 for details of the three original tests and the subsequent modifications used in this study).

#### Swabbing of glass plates and tubes

A two-swab technique (wet and dry) using 155C Copan^©^ swabs (Interpath, Victoria, Australia) was used across all relevant tests.

### Test 1: Deposition of a handprint on a glass plate

#### Sample deposit

Fifteen minutes post-handwashing, participants were asked to place their dominant hand on a cleaned glass plate (140 × 220 mm; 4 mm thickness) for 10 s, with their fingers and thumb together, applying firm pressure but no friction, as adapted from Goray & van Oorschot [15] (See Supplementary Data 3 for detailed experimental conditions used). The side of the glass plates on which the deposit was made were swabbed in their entirety. Both the clean and dirty conditions were conducted in triplicate for each participant. Each of the deposits were made on a different day.

#### DNA analysis

For Tests 1 and 2 (Section 2.3), DNA was extracted from both swabs together with the DNA IQ system (Promega, Madison, WI, USA) (end volume 60 µL), quantified using the Quantifiler® Trio DNA Quantification Kit on an ABIPRISM® 7500 (Life Technologies, Carlsbad, CA, USA), and amplified using PowerPlex® 21 (Promega, Madison, WI, USA) (maximum template input of 0.5 ng in a maximum volume of 15 µl, 25 µl total volume, 30 cycles). PCR products were then separated on a 3500 Genetic Analyser (Life Technologies, Carlsbad, CA, USA) and analysed using GeneMapper® ID-X software (v1.6, Life Technologies, Carlsbad, CA, USA) with a detection threshold of 175 RFU. Note, all samples were submitted to amplification, independent of detected DNA concentration.

#### Result interpretation

The minimum number of contributors (MNC) was determined for each profile. The total number of alleles, allelic peak heights, and mixture proportions was used to inform the number of contributors. The homozygosity threshold was set at of 2,000 RFU. The total amount of DNA was calculated by multiplying the DNA quantification result (in ng/μL) by the volume of an extract (60 μL). This total DNA amount was divided into donor and non-donor contributions using the mixture proportions generated by STRmix™ (v2.9, ESR, New Zealand and FSSA, South Australia).

The average RFU of the donor alleles was calculated by dividing the donor’s total RFU contributions by the total number of alleles detected, excluding Amelogenin. STRmix™-generated mixture proportions were further used to assign major and minor contributors, as applicable, where the major contributor deposited over 70% of the total DNA in a mixed sample. Where a major donor couldn’t be established, majority and minority contributors were defined. DNA profiles were deconvoluted in STRmix™ and compared to reference profiles from the participants, investigator, and other users of the shared laboratory space to identify relevant contributors.

####  Shedder categorisation

Goray & van Oorschot [15] described five shedder status allocations where participants were evaluated in terms of total DNA, number of alleles, and relative fluorescence units (RFU) per deposit. Additional factors considered included mixture proportions, as well as a participant’s tendency to be the major contributor within a mixture. Ultimately, participants could be described as low, low-intermediate, intermediate, intermediate-high, or high shedders. For the purposes of this study, the exact results described in Goray & van Oorschot [15] were used to generate the cut-off ranges used for categorisation. We recognise that the ranges described were based off data collected from 10 participants and a larger sample size may have resulted in different cut-off values, as is the case with most investigations into shedder categorisation. Participants were placed into one of the five shedder categories (Table 1) based on thresholds met in at least two of the following three categories: total DNA, number of alleles, and average RFU. Additionally, to be classified as an intermediate-high or high shedder, participants had to always be the major contributor in a mixture, contributing 70% or more of the DNA collected in each sample deposited. Goray and van Oorschot’s [15] categorisation method was applied to DNA quantity and quality data obtained from samples generated using both Test 1 and Test 2 deposition methodologies.

**Table 1:** Shedder status criteria as used by Goray & van Oorschot [15]

Designation	Donor DNA Deposited (ng)	Average Donor RFU	Number of Donor Alleles (%)	Donor’s Mixture Proportions *(as per STRmix*^*TM*^*)*	Always major contributor?
Low	0.009–0.1	245–522	18–42	44–72	No
Low-Intermediate	0.07–0.2	314–893	43–63	58–83	No
Intermediate	0.12–0.52	541–1,700	70–88	80–91	Yes
Intermediate-High	0.25–1.43	1231–3,030	84–92	60–96	Yes
High	4.57–4.98	4,163–4,708	99–100	92–96	Yes

### Test 2: Deposition of a grip mark on a plastic conical tube

#### Sample deposit and DNA analysis

Fifteen minutes post-handwashing, participants were instructed to grip a cleaned plastic conical tube (Greiner centrifuge tube, 50 mL, 30 × 115 mm) with firm pressure for 10 s using their dominant hand, as adapted from Fonneløp et al. [18]. Participants were asked to ensure that their entire hand spanned the body of the tube and that no part touched the cap. The body of each plastic tube was swabbed, while the caps remained unsampled. Both the clean and dirty conditions were conducted in triplicate for each participant. Each of the deposits were made on a different day. The DNA analysis of these samples was the same as for the Test 1 samples described in the "DNA Analysis" Section.

#### Shedder categorisation

Fonneløp et al. [18] used a binary system of classification, which only recognised individuals as high or poor shedders (see Supplementary Data 3 for more detail). Participants were classified as high shedders if at least two of their three deposits produced a higher than average (for all tested participants) DNA quantification result and a high-quality profile with 12 or more full donor loci. All other participants were categorised as poor shedders. The average thresholds were determined using data generated by our six test participants. The following modification was adopted in this study: Fonneløp’s analysis utilised PowerPlex^(R)^ 17, which analyses alleles at a total of 17 loci, including Amelogenin, where 12/17 complete loci represents approximately 71% coverage. The present study utilised PowerPlex^(R)^ 21, assessing 21 loci inclusive of Amelogenin. To account for the increased number of typed loci, a similar 71% cut-off was applied, and a minimum of 15 full donor loci was required for a high shedder status designation. The Test 2 categorisation method was applied to quantity and quality data obtained from samples generated after applying Test 1 and Test 2 deposition methodologies.

An additional “modified Fonneløp et al.” categorisation method, as first described in Goray et al. [39], was applied to the cell counts generated in Test 3. Cell scores were averaged for all participants in the clean and dirty conditions and participants were deemed high shedders if at least two of their three deposits produced a higher than average number of cells for said condition. This allowed for the binary categorisation of Test 3 results and subsequent comparison to Tests 1 and 2 results.

### Test 3: Deposition of a thumbprint on a glass slide

#### Sample deposit

As the thumb was not involved in the tube gripping manoeuvre (as per instructions to participants), Test 3 was conducted immediately after Test 2. Participants were asked to place their dominant thumb on a cleaned DNA-free glass slide with medium pressure for 15 s, as adapted from Kanokwongnuwut et al. [11] (see Supplementary Data 3 for more detail). Both the clean and dirty conditions were run in triplicate for each participant. Each of the deposits were made on a different day.

#### Slide staining

Slides were stained with 10 μL of a 20-fold dilution of the Diamond™ Nucleic Acid Dye (Promega, Madison, WI, USA) stock (10,000x). The dye was left to dry for 30 min before slides were visualized under a Dinolite fluorescent digital microscope (Dino-Lite Australia) equipped with an emission filter of 510 nm and a blue LED excitation light source (480 nm).

#### Cell scoring

Cells were manually scored by counting the number of “bright spots” or cells in three frames, each 1 mm^2^ in size, at 220 × magnification. Each of these frames was thought to represent a different “high-density” area of the thumbprint, as first conducted by Kanokwongnuwut (personal communication). The cell counts within these frames were averaged to produce an overall cell count per deposit.

#### Shedder categorisation

Kanokwongnuwut et al. [11] recorded their 11 donors as being either high, intermediate, or low shedders, albeit recognising that shedder status is best represented by a continuum. While the original study utilised RFU rather than cell scores for the purposes of categorisation, cell ranges were extracted from the figure titled “Illustrating the amount of cell nuclei deposited at four different time points” [11], where deposits of 1–15 cells/mm^2^ were made by low shedders, deposits of 16–30 cells/mm^2^ were made by intermediate shedders and deposits of more than 30 cells/mm^2^ were made by high shedders. Although these ranges may not have been intentioned for use by the authors, these values have been the basis of subsequent shedder studies [12, 34]. As such, these ranges were used for the purposes of designation in Test 3. A correlation between cell scores and RFU generated (R^2^ = 0.92175) was found for 9/11 participants in Kanokwongnuwut et al. [11], with the outliers being two intermediate shedders.

#### Evaluation of complete fingermarks

To further evaluate different shedder methods and the effects of categorisation criteria, the fingermarks generated in Test 3 were overlayed with a 5 mm^2^ grid and images of every square with at least 50% cell coverage, regardless of density, was taken at 50 × magnification, as modified from Kaesler et al. [34]. ImageJ (LOCI, University of Wisconsin) was used to perform automated cell counts on each of these 5 mm^2^ frames. To accurately compare these results to the Test 3 designations, a modified Kanokwongnuwut et al. [11] method was used. Three 5 mm^2^ frames of high cell density were selected and the generated cell counts were averaged and divided by 25 to account for differences in grid size used (5 mm^2^ versus 1 mm^2^ in Test 3). Two other methods of classification were applied, including modified Fonneløp et al. [18] (as described in the ''Shedder Categorisation'' Section) and an in-house, “sample-adjusted” method. In this sample-adjusted method, for each condition, all participant replicate results were ranked from lowest to highest. These results were then divided into equal thirds, each of which represented the accepted ranges for a low, intermediate, and high shedder, respectively. In the instance that a specific value spanned two categories, it was conservatively estimated to be the upper limit of the lower ranked shedder category. A total of 665 squares were counted over the entire study. The resulting shedder designations are presented in Table 7.

### Shedder categorisation terminology

The terminology used to describe similar shedding patterns vary between publications, as some will use good/high or bad/poor/low shedder terms interchangeably (see Supplementary Data 1). In this article, we used high, intermediate, and low shedder terms.

### Quality controls and cleaning protocols

To ensure the glass plates and plastic conical tubes were DNA-free prior to use, they were sprayed in sequence with 1% hypochlorite, 100% ethanol, and distilled water. Both surfaces were dried with paper towel after each solvent was applied and each item was individually stored in plastic bags. Control swabs were taken from 15 random glass plates and plastic conical tubes after cleaning. No quantifiable DNA and no DNA profiles were generated from control samples. Both positive and negative controls were appropriately implemented during DNA processing and produced the expected results.

The glass slides used were cleaned with 100% ethanol. Once deposits were made, the slides were stored in a cupboard until staining to prevent cell degradation due to environment [40]. Negative control slides (10) were identically cleaned, stored, and stained on random sampling dates. No cells were observed on these controls.

### Statistical analysis

Given the continuous nature of the variables assessed, a Mann–Whitney U-test was used to analyse the differences in DNA deposits (ng) made between Tests 1 and 2, as well as between the clean and dirty hands conditions (*p* < 0.05 significance). Inter-person variation was also assessed for each test conducted. All statistical analyses were performed in SPSS Statistics (IBM, v29.0.1.0).

## Results

### General

Of the 72 samples generated from Tests 1 and 2, 11 failed to generate a DNA profile (15%). Each of these samples produced a very low (≤ 0.06 ng) (18%) or negative (82%) DNA quantification result. Of note, nine of these samples were from the clean hands condition, which suggests insufficient DNA re-accumulation in the 15-min wait period.

In the remaining samples that generated a profile, a depositor was detected in 58 (95%) samples (LRs of inclusion: 1.0e^0^—4.6e^26^). Conversely, likelihood ratios produced by STRmix™ determined that the participant or “donor” did not contribute DNA to deposits made in three of these samples, as only non-self-DNA was detected. All non-donors were assigned an “unknown” designation.

### Test 1: Deposition of a handprint on a glass plate

#### DNA quantity

On average, the total quantity of DNA recovered from a single deposit onto a glass plate ranged from 0–0.3 ng (average 0.08 ng) in the clean condition and 0–0.56 ng (average 0.18 ng) in the dirty condition. The differences in deposition seen between conditions was statistically significant (*p* = 0.016). Individual participant results are outlined in Table 2. Based on these quantification results, no high shedders were identified.

**Table 2:** Test 1 replicate results for all participants, as adapted from Goray & van Oorschot [15], in the clean condition and dirty hands conditions.  The number of contributors per replicate are denoted and all non-donors were considered “unknowns” for the purpose of analysis. To be named the major donor, an individual must have contributed 70% or more of the total DNA generated within a mixture. The donor likelihood ratios are provided in the table

		Number of Contributors		Donor DNA (ng)		Donor Alleles (%)		Donor Average RFU		Mixture Proportions (Donor: Non-Donor)		Donor Major Contributor		Likelihood Ratio	
Participant	Replicate	Clean	Dirty	Clean	Dirty	Clean	Dirty	Clean	Dirty	Clean	Dirty	Clean	Dirty	Clean	Dirty
1	1	N/A^a^	N/A	0	0	0	0	0	0	N/A	N/A	N/A	N/A	N/A	N/A
	2	N/A	1	0.06	0.12	0	3	0	309	N/A	100:0	N/A	Yes	N/A	3.2e^0^
	3	1	1	0.06	0.06	5	10	202	264	100:0	100:0	Yes	Yes	4.2e^1^	9.2e^0^
	**Average**	1	1	0.04	0.06	2	4	67	191						
2	1	2	2	0.21	0.54	38	90	240	622	71:29	91:9	Yes	Yes	8.5e^6^	4.8e^22^
	2	1	1	0.3	0.24	30	35	405	397	100:0	100:0	Yes	Yes	1.8e^8^	5.1e^9^
	3	2	2	0	0.24	0	67	0	454	0:100	69:31	No	No	2.7e^−1^	2.0e^13^
	**Average**	2	2	0.17	0.34	23	64	215	491						
3	1	N/A	1	0	0.12	0	18	0	220	N/A	100:0	N/A	Yes	N/A	1.4e^4^
	2	1	1	0.06	0.12	10	5	230	315	100:0	100:0	Yes	Yes	7.5e^1^	2.3e^0^
	3	1	2	0.06	0.12	8	23	206	308	100:0	67:33	Yes	No	3.9e^1^	5.4e^2^
	**Average**	1	1	0.04	0.12	6	15	145	281						
4	1	1	2	0.12	0.56	13	68	255	758	100:0	93:7	Yes	Yes	1.8e^2^	2.3e^17^
	2	N/A	2	0	0.16	0	25	0	273	N/A	68:32	N/A	No	N/A	6.7e^3^
	3	1	1	0.12	0.18	13	25	255	392	100:0	100:0	Yes	Yes	3.2e^2^	7.5e^6^
	**Average**	1	2	0.08	0.3	9	39	170	474						
5	1	2	2	0.06	0.25	15	45	448	256	31:69	70:30	No	Yes	1.5e^0^	3.2e^10^
	2	N/A	2	0	0.15	0	28	0	200	N/A	63:37	N/A	No	N/A	2.8e^3^
	3	N/A	2	0	0.2	0	50	0	215	N/A	65:35	N/A	No	N/A	1.6e^10^
	**Average**	1	2	0.02	0.2	5	41	149	224						
6	1	2	N/A	0.13	0.06	15	0	212	0	70:30	N/A	Yes	N/A	5.8e^2^	N/A
	2	1	2	0.06	0	3	0	254	0	100:0	0:100	Yes	No	2.1e^0^	9.8e^−1^
	3	1	1	0.24	0.18	50	38	357	524	100:0	100:0	Yes	Yes	5.6e^13^	1.2e^12^
	**Average**	1	1	0.14	0.08	23	13	274	175						
**Overall Participant Averages**		**1**	**2**	**0.08 ng**	**0.18 ng**	**11% alleles**	**29% alleles**	**170 RFU**	**306 RFU**						

^a^“N/A” results are reflective of samples that did not generate a DNA deposit and/or profile

Participants 2 and 5 were both classified as low shedders in the clean condition and as low-intermediate shedders in the dirty condition. All other participants were classified as low shedders in both conditions. There was significant inter-person variation in DNA deposits made by participants in the dirty (*p* = 0.046), but not the clean (*p* = 0.313) condition.

Generally, participants deposited more DNA in the dirty hands condition relative to the clean, with the exception of Participant 6, who consistently deposited more DNA in the clean condition, an outlier result that cannot be explained by reported pre-deposit activities.

#### DNA profiling analysis

Most samples (15/28, 53.5% total samples) generated single source profiles, while the rest consisted of two-person mixtures (see Table 2). Notably, four of the samples deposited in the clean hands condition were two-person mixtures, relative to the nine two-person mixtures deposited in the dirty hands condition. The number of alleles deposited, excluding Amelogenin, ranged from 0–20 in the clean condition (average 4 alleles) and 1–36 in the dirty condition (average 12 alleles). Additionally, the donor RFU deposited ranged from 0–405 in the clean condition (average 176) and 0–758 in the dirty condition (average 306). Individual participant results are outlined in Table 2. In the 13 mixtures generated, on average, the major contributor deposited 70% and 72% of the total DNA generated in the clean and dirty hands conditions, respectively. The donor was the major contributor in 5/13 mixtures (38%) and the majority contributor in an additional five mixtures. An unknown non-donor was the major contributor in one mixture (Participant 6, Test 1, Replicate 2, Dirty Hands) and the majority contributor in one mixture (Participant 5, Test 1, Replicate 1, Clean Hands). In the last two-person mixture, generating only four alleles (Participant 2, Test 1, Replicate 3, Clean Hands), Participant 2 was excluded as a contributor.

#### Pre-deposit activity analysis

Data gained from the circulated surveys did not identify any clear trends impacting the quality and quantity of deposits (see Supplementary Data 4 for activities performed by each participant during each given wait period). In the dirty hands condition, most participants spent their wait periods sitting in their personal or shared office, doing office work, using their phones, and talking to their peers at an unknown distance. Half the participants reported touching their faces briefly during the wait period, but no observable differences were detected in deposits made. Throughout both conditions, all participants refrained from nail-biting, coughing, or sneezing into one’s hands. Furthermore, all participants wore gloves at least once in the four hours prior to washing their hands and depositing a sample, but no significant differences in deposits were seen.

#### Shedder criteria comparison

To assess the impact of categorisation criteria, the criteria taken from Fonneløp et al. [18] was applied to Test 1. The averaged results for each group of replicates are outlined in Table 3. The inclusion of criteria that recognises an intermediate subgroup of shedders, as used in Test 1, resulted in two participants meeting the threshold for low-intermediate status, suggesting that low shedders, as defined by a binary classification system, may be more accurately described as low-intermediate shedders.

**Table 3:** A comparison of shedder status categorisations for all participants from Test 1 using criteria adapted from Goray & van Oorschot [15] and Fonneløp et al. [18]

	Handprint on a Glass Plate: as per Goray & van Oorschot’s criteria [15]		Handprint on a Glass Plate: as per Fonneløp et al.’s criteria [18]	
Participant	Clean	Dirty	Clean	Dirty
1	Low	Low	Low	Low
2	Low	Low-Intermediate	Low	Low
3	Low	Low	Low	Low
4	Low	Low-Intermediate	Low	Low
5	Low	Low	Low	Low
6	Low	Low	Low	Low

### Test 2: Deposition of a grip mark on a plastic conical tube

#### DNA quantity

On average, the total quantity of DNA recovered from a single deposit onto a plastic conical tube ranged from 0–3 ng (average 0.26 ng) in the clean condition and 0.04–2.43 (average 0.44 ng) in the dirty condition. The differences seen between conditions were statistically significant (*p* = 0.017). Individual participant results are outlined in Table 4.

**Table 4:** Test 2 replicate results for all participants, as adapted from Fonneløp et al. [18]

		Number of Contributors		Donor DNA Deposited (ng)		Number of Full Donor Loci		Number of Donor Alleles	
Participant	Replicate	Clean	Dirty	Clean	Dirty	Clean	Dirty	Clean	Dirty
1	1	2	2	0.04	0.12	1	1	6	5
	2	N/A	1	0	0.06	0	0	0	3
	3	1	1	0	0.24	0	4	1	12
	**Average**	1	1	0.01	0.14	0	2	2	7
2	1	2	1	0.35	0.24	15	8	32	19
	2	1	2	0.06	2.43	1	19	6	38
	3	1	2	3	1.80	21	20	40	39
	**Average**	1	2	1.14	1.49	12	16	26	32
3	1	2	2	0.08	0.13	6	6	19	21
	2	2	2	0.04	0.22	0	9	2	30
	3	1	1	0.06	0.12	1	5	2	16
	**Average**	2	2	0.06	0.16	2	7	6	22
4	1	1	2	0.3	0.04	6	3	18	17
	2	2	1	0.17	0.6	1	5	10	20
	3	1	2	0.12	0.17	0	1	3	21
	**Average**	1	2	0.20	0.27	2	3	10	19
5	1	1	2	0	0.12	0	1	0	5
	2	1	3	0.24	0.09	0	6	2	16
	3	N/A	2	0	1.07	0	21	0	40
	**Average**	1	2	0.08	0.43	0	9	1	20
6	1	2	1	0.11	0.06	7	0	20	1
	2	N/A	1	0	0.12	0	5	0	17
	3	1	1	0.06	0.3	0	8	7	21
	**Average**	1	1	0.06	0.16	2	4	9	13
**Overall Participant Average**		**1**	**2**	**0.26**	**0.44**	**4**	**7**	**9**	**18**

Participant 2 deposited the highest average amount of DNA in both conditions and was consequently the only participant to meet the DNA threshold necessary for a high shedder designation. All other participants were determined to be low shedders in both conditions. Inter-participant variation was not significant in either the clean (*p* = 0.092) or dirty (*p* = 0.371) conditions.

#### DNA profiling analysis

Samples generated were either single source (51.5% total samples) profiles, two-person (45.5% total samples), or three-person mixtures (3% total samples) (see Table 4). The number of full loci deposited ranged from 0–21 in both conditions (clean average: 4, dirty average: 7). However, again, only Participant 2 met the loci-specific criterion required for a high shedder designation.

#### Pre-deposit activity analysis

The distribution of pre-deposit factors remained similar to that found in Test 1, as samples continued to be taken at similar times throughout the workday. None of the aforementioned observable factors produced a noticeable effect on DNA deposits (see Supplementary Data 4).

#### Shedder criteria comparison

To assess the impact of categorisation criteria, Goray & van Oorschot’s [15] criteria was applied to Test 2 samples. The averaged results for each group of replicates are outlined in Table 5.

**Table 5:** A comparison of shedder status designations for all participants from Test 2 using criteria adapted from Fonneløp et al. [18] and Goray & van Oorschot [15]

	Grip Mark on a Plastic Conical Tube as per Fonneløp et al.’s criteria [18]		Grip Mark on a Plastic Conical Tube as per Goray & van Oorschot’s criteria [15]	
Participant	Clean	Dirty	Clean	Dirty
1	Low	Low	Low	Low
2	High	High	Intermediate-High	Intermediate-High
3	Low	Low	Low	Low-Intermediate
4	Low	Low	Low-Intermediate	Low-Intermediate
5	Low	Low	Low	Low-Intermediate
6	Low	Low	Low	Low

When Goray et al.’s [15] categorisation method was applied to the clean condition results generated in Test 2 (see Table 5 or Supplementary Data 5 for the complete data set) and the subsequent shedder status designations were compared, Test 2 generated higher shedder designations in 55.5% (10/18) of the samples, lower shedder designations in 11.1% (2/18) of the samples, and the same designations in the remaining samples (6/18). In the dirty condition, Test 2 generated replicates with higher shedder designations in 61.1% (11/18) of the samples, lower shedder designations in 16.6% (3/18) of the samples, and the same designations in the remaining samples (4/18). Although deposits that failed to generate a quantifiable result or subsequent DNA profile post-sampling were classified as low shedder deposits, for the purposes of comparison between Tests 1 and 2, samples that generated no result were considered “lower” than samples that deposited identifiable, but low amounts of DNA or allelic content.

### Test 3: Deposition of a thumbprint on a glass slide

#### Cell scores

**Fig. 1:**
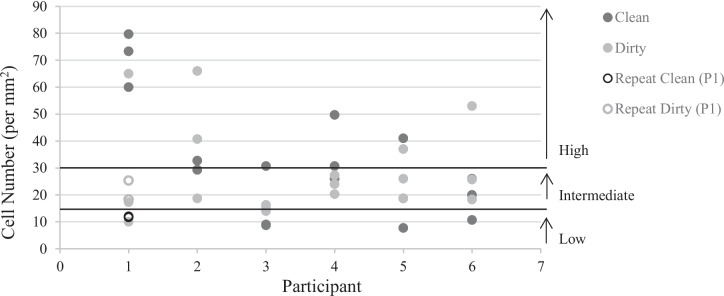
Test 3 replicate results for all participants as adapted from Kanokwongnuwut et al. [11], including the four additional or “repeat” samples from Participant 1. Ranges for low, intermediate, and high shedders are also highlighted

The number of cells deposited onto three 1 mm^2^ frames on a glass slide ranged from 9–80 (average 32 cells) in the clean condition and 10–65 (average 29 cells) in the dirty condition. The difference seen between conditions was not statistically significant (*p* = 0.447). Individual participant results, including the four additional samples (two clean, two dirty) taken from Participant 1 twelve months post-initial deposits, are outlined in Fig. 1.

Participants 1 and 2 were categorised as high shedders, while Participants 3 and 5 were intermediate shedders in both test conditions. Participant 4 was a high shedder in the clean condition, but an intermediate shedder in the dirty hands condition. Comparatively, Participant 6 was an intermediate shedder in the clean condition, but a high shedder in the dirty condition. No low shedders were identified in this test group. Inter-participant variation was not significant in the clean (*p* = 0.065) or dirty hands (*p* = 0.209) condition.

#### Shedder criteria comparison

To assess the impact of categorisation criteria, a modified cell-counting method adapted from Fonneløp et al.’s [18] was applied to the scores generated in Test 3. The average of all cell counts across all participants was calculated and if at least 2/3 replicates surpassed this average, they were classified as high shedders. All other participants were deemed low shedders. Individual participant results are outlined in Table 6.

**Table 6:** A comparison of shedder status designations for all participants from Test 3 using criteria from Kanokwongnuwut et al. [11] and criteria adjusted from Fonneløp et al. [18]

	Fingermark on a DD-Stained Slide as per Kanokwongnuwut et al. [11]		Fingermark on a DD-Stained Slide as adapted from Fonneløp et al.[18]			
Participant	Clean	Dirty	Clean		Dirty	
			Average Cells Scored	Designation	Average Cells Scored	Designation
1	High	High	71	High	30.8	Low
2	High	High	30	Low	41.8	High
3	Intermediate	Low	16	Low	15.1	Low
4	High	Intermediate	36	Low	23.9	Low
5	Intermediate	Intermediate	23	Low	27.2	Low
6	Intermediate	High	19	Low	32.3	Low

This change in criteria noticeably impacted classification, as all intermediate shedders became low shedders, and in four instances, participants transitioned from high to low shedder status, with these occurrences being evenly split between the clean and dirty hands conditions. No participants transitioned from low to high shedder status.

The complete fingermarks generated as part of Test 3 were imaged at 50 × magnification and scored using ImageJ. Results were subsequently categorised using shedder criteria modified from Kanokwongnuwut et al. [11], Fonneløp et al. [18], as well as an in-house sample-adjusted method (described in  the "Evaluation of Complete Fingermarks" Section).

Kaesler et al. [34] and Goray et al. [39] previously encouraged analysis of entire fingermarks rather than select 1 mm^2^ frames, as cellular material is non-uniform along the finger surface [34] and discrepancies in shedder status may reflect frame selection rather than inherent deposition tendencies. As the fingermarks generated in Test 3 allowed for further analysis, cell scores of the entire fingermark were completed and multiple sets of categorisation criteria were applied to these results, as well as those generated in Test 3. These relative shedder outcomes are highlighted in Table 7.

**Table 7:** Resultant shedder status category designations for all participants as modified from Kaesler et al. [34] for both the clean and dirty hands conditions

	Test 3		Complete Fingermark		Test 3		Complete Fingermark		Test 3		Complete Fingermark	
	Designation as per Kanokwongnuwut et al. [11]		Designation as per modified Kanokwongnuwut et al. [11]		Designation as per modified Fonneløp et al. [18]				Designation as per sample-adjusted method			
Participant	Clean	Dirty	Clean	Dirty	Clean	Dirty	Clean	Dirty	Clean	Dirty	Clean	Dirty
1	High	High	Low	Low	High	Low	High	High	High	High	High	Int
2	High	High	Low	Low	Low	High	Low	High	High	High	Int	High
3	Int	Low	Low	Low	Low	Low	Low	Low	Low	Low	Low	Low
4	High	Int	Low	Low	Low	Low	High	High	High	Int	High	Intermediate
5	Int	Int	Low	Low	Low	Low	Low	Low	Low	High	Low	Int
6	Int	High	Low	Low	Low	Low	Low	Low	Low	High	Int	Int

When the shedder ranges suggested by Kanokwongnuwut et al. [11] were applied to the entire fingermark, all participants were low shedders in both conditions, relative to Test 3 where no persistently low shedders were seen. Applying modified Fonneløp et al. [18] to Test 3 data, 33% (2/6) and 50% (3/6) of participants were high shedders in the clean and dirty condition, respectively. When this categorisation criteria was applied to the entire fingermark, 66% (4/6) of participants maintained their shedder status in both conditions while Participants 1 and 4 transitioned from a low to high shedder status in at least one condition. The in-house, sample-adjusted method used participant generated cell scores to create the cell ranges for low, intermediate, and high shedders. Using this criteria, an equal third of participants were low, intermediate, and high shedders in the clean condition. In the dirty condition, 67% (4/6) of participants became intermediate shedders, while 17% of participants (1/6) were low and high shedders, respectively.

### Comparing shedder category status designations

The shedder category status designation for each of the 108 deposits made is listed in Table 8, as assessed by the criteria outlined in each of the original studies [11, 15, 18]. The average shedder status designation is subsequently denoted in Table 9.

**Table 8:** Shedder status category designations for all participant replicates across all three tests, as defined by the criteria outlined in the original studies [11, 15, 18]

		Test 1: Handprint on a Glass Plate	Test 2: Grip Mark on a Plastic Conical Tube	Test 3: Fingermark on a DD-Stained Slide	Test 1: Handprint on a Glass Plate	Test 2: Grip Mark on a Plastic Conical Tube	Test 3: Fingermark on a DD-Stained Slide		
Participant	Replicate	Clean	Dirty	Clean	Dirty	Clean	Dirty		
1	1	ND^a^	ND	Low	Low	High	Intermediate		
	2	NP^b^	Low	ND	Low	High	High		
	3	Low	Low	Low	Low	High	Low		
2	1	Low	Intermediate-High	High	Low	High	Intermediate		
	2	Low-Intermediate	Low-Intermediate	Low	High	Intermediate	High		
	3	NP	Low-Intermediate	High	High	Intermediate	High		
3	1	ND	Low	Low	Low	Low	Low		
	2	Low	Low	Low	Low	Low	Intermediate		
	3	Low	Low	Low	Low	High	Low		
4	1	Low	Intermediate	Low	Low	Intermediate	Intermediate		
	2	ND	Low	Low	Low	High	Intermediate		
	3	Low	Low	Low	Low	High	Intermediate		
5	1	Low	Low-Intermediate	NP	Low	High	Intermediate		
	2	ND	Low	Low	Low	Low	High		
	3	ND	Low-Intermediate	ND	High	Intermediate	Intermediate		
6	1	Low	NP	Low	Low	Intermediate	Intermediate		
	2	Low	NP	ND	Low	Low	Intermediate		
	3	Low-Intermediate	Low-Intermediate	Low	Low	Intermediate	High		

^a^“ND” indicates a sample where no DNA was quantified and no subsequent DNA profile was generated

^b^“NP” indicates a sample where DNA was quantified but no subsequent donor DNA profile was generated

**Table 9:** Shedder status category designation for each participant across all three tests [11, 15, 18] as averaged from the replicates in Table 8

	Test 1: Handprint on a Glass Plate		Test 2: Grip Mark on a Plastic Conical Tube		Test 3: Fingermark on a DD-Stained Slide	
Participant	Clean	Dirty	Clean	Dirty	Clean	Dirty
1	Low	Low	Low	Low	High	High
2	Low	Low-Intermediate	High	High	High	High
3	Low	Low	Low	Low	Intermediate	Low
4	Low	Low-Intermediate	Low	Low	High	Intermediate
5	Low	Low	Low	Low	Intermediate	Intermediate
6	Low	Low	Low	Low	Intermediate	High

As outlined in Table 8, within Test 1, where five shedder designations were possible, Participants 1 and 3–5 maintained their low shedder status between clean replicates, while the remaining participants transitioned between low and low-intermediate status. In the dirty condition, Participants 1 and 3 maintained their low shedder status between replicates, Participants 4–6 transitioned between low and low-intermediate, and Participant 2 transitioned between low-intermediate and intermediate-high categories.

Within Test 2, where only low and high designations were possible, Participants 1, 3, 4 and 6 maintained their low shedder status in both conditions. Participant 5 made one deposit in the dirty condition that met the criteria for a high shedder designation but was otherwise a low shedder in both conditions. Comparatively, Participant 2 transitioned between low and high shedder designations in both conditions.

Within Test 3, where low, intermediate, and high shedder designations were possible, no participants maintained their shedder status designation between replicates in either condition. Participants 2 and 4 transitioned between intermediate and high designations, whereas all other participants transitioned between all three categories.

As outlined in Table 9, Test 3 generated higher status replicates relative to Test 1 in 77.8% (14/18) of clean samples and consistent results in the remaining samples (4/18). Relative to Test 2, in the clean condition, Test 3 generated higher status replicates in 61.1% (11/18) of samples, lower status replicates in 5.5% (1/18) of samples, and consistent results in the remaining samples (6/18). Findings were largely consistent between the clean and dirty hands conditions. In the dirty condition, when Test 3 was compared to Test 1, participants generated higher status replicates in 61.1% (11/18) of samples and maintained their status in the remaining samples (7/18). When Test 3 was compared to Test 2, participants generated higher status replicates in 61.1% (11/18) of the dirty samples, lower status replicates in 11.1% (2/18) of samples, and consistent results in the remaining samples (5/18).

## Discussion

### Comparing shedder designations

In this study, we investigated whether six individuals maintained their shedder status category designation between three different, well-recognised shedder tests. We attempted to further standardise these methods by implementing a handwashing regimen, imposing pre-deposit activity restrictions, and utilising the same sampling technique; however, differences in what was being compared and the criteria used to define shedder status impacted resultant shedder status designations.

When the data from participants were separated by donor, test, and testing condition (clean or dirty hands), no significant inter-personal variation was found within this cohort, which likely reflects the incidental over-inclusion of low shedders.

Five participants maintained their low (or low-intermediate) shedder status between Tests 1 and 2, while Participant 2, who was a low-intermediate shedder within Test 1, was the only participant to meet the DNA and complete loci thresholds for a high-status designation within Test 2. When Goray & van Oorschot’s criteria [15] was applied to Test 2 samples, Participant 2’s status fell to intermediate-high in both conditions, suggesting that a more expansive classification system may more accurately classify both “low” and “high” shedders. Additionally, when intermediate shedders were re-classified within a binary system, they consistently became low shedders as they couldn’t surmount the threshold set for high shedders and instead fell into the “catch all” of remaining low shedders.

Comparatively, all participants were categorised as either an intermediate or high shedder in one, if not both conditions, when Test 3 was utilised. Within our sample, Participant 1 varied the most between tests, as they were the lowest tested shedder throughout Tests 1 and 2, but the highest shedder using Test 3. To determine whether these initial fingermarks were outlier deposits, Participant 1 was retested one year later. In these additional samples, Participant 1 deposited an average of 12 and 22 cells in the clean and dirty hands conditions, respectively, resulting in a low and intermediate shedder status designation as per Kanokwongnuwut et al. [11]. There were no substantial differences in pre-deposit activities when compared to the previous year, which suggests that although participants may maintain their shedder status over time when DNA deposition and analysis is utilised [15], there may be more variability with cell deposition and scoring, which should be reflected in the number of replicates used.

### Comparing DNA quantification to cell scores

When comparing DNA quantifications to cell scores, it’s impossible to determine whether cell scores regularly over-estimate shedder status or if, alternatively, DNA quantification and analysis consistently underestimates shedder status. Although the grip and fingermarks were made by participants consecutively, suggesting that any impact of internal or external participant factors would be consistent between tests, the amount of DNA deposited in Test 2 could only be weakly correlated with the cell deposits generated in Test 3 (R^2^ = 0.002 in the clean condition and R^2^ = 0.25 in the dirty condition).

We’ve shown that shedder status varies between different tests and there are numerous potential interacting factors as to why. Compared to cell scores, DNA quantification allows for the identification of individuals in a mixture, which may prevent over-estimations of shedder status. However, DNA loss associated with sampling, extraction, and STR typing may range from 20–80% [41, 42], is difficult to predict [11], and is largely unaccounted for [43, 44] in subsequent analysis. These factors can only be mitigated, in part, by ensuring that different laboratories use identical methods, including downstream processing of samples.

Furthermore, when considering fingermarks, corneocytes exist on a non-uniform degradation continuum, containing variable amounts of DNA, if any, along the entire fingermark [28, 34, 41]. Additionally, DD cannot effectively illuminate cell-free DNA, which may comprise approximately 60% of the total DNA generated from a touch deposit [7, 41], as these components are not visible at a magnification of 220x [34]. Goray et al. [39] recently considered the impact of observer bias in manual cell scores, as scores of the same deposit could differ by 0–75% between different observers or even, the same observer at different timepoints. The use of counting software, such as ImageJ^©^, helped minimise this discrepancy, but the ideal program inputs remain unknown and requires further testing. Ultimately, this unknown variability in deposits makes comparing the results of different shedder tests unfeasible.

### Quantity and quality of DNA samples

Within this study, DNA recovery fell into a limited range of 0–0.56 ng in Test 1 and 0–3 ng in Test 2, inclusive of both conditions. This small deposit range, relative to previous studies [17, 29, 45], may reflect the compounded effects of regimented handwashing, sample size, and differences in the efficiencies and thresholds of the methodologies applied (from sample collection, DNA quantitation, DNA amplification through to profile analyses) during downstream processing. Moreover, of these samples, only 3% generated full profiles and 82% generated partial profiles, which increases the impact of stochastic effects, as seen in three samples where only non-self-DNA was identified in samples generating four or less alleles.

### Impact of handwashing and activity

The effect of handwashing on DNA deposition is controversial, as there’s no clear-cut relationship between handwashing and subsequent cells shed [2, 12, 21]. In the clean condition, an individual’s inherent ability to shed cells, or their “true” shedder status, was assessed, while in the dirty condition, the deposits made also reflected DNA accumulation from contacts made with their environment, which better represents real world circumstances [20, 46]. In Tests 1 and 2, significant differences in DNA deposition were seen between the clean and dirty conditions. This suggests that shedder status, as determined from a washed hand, may be different to what is determined from “dirty” samples, as the manner of handwashing and the activities conducted would impact the amount and sources of DNA deposited. Considering the activity restrictions placed on participants during the dirty condition, differences in deposition trends may be even larger than that observed in this study. As such, further insight into deposit variability as seen in samples with and without pre-deposit restrictions is desirable.

Comparatively, no significant difference was seen between conditions in Test 3, possibly because donor cells cannot be distinguished from non-donor cells within a DD-stained fingermark. Although handwashing is meant to limit the overall impact of previously accumulated cells or exogenous DNA, within Tests 1 and 2, the number of observed mixtures only fell to 25% in the clean condition (from 53% in the dirty condition), which suggests that handwashing is not sufficient in removing all exogenous DNA, particularly when you consider individual differences in handwashing technique. Thus, a method that does not utilise mixture proportions may not be sensitive to differences in a clean relative to dirty condition. As “dirty” or mixed samples are more reflective of everyday deposits, including those made at crime scenes, the ability to confirm the presence of specific contributors increases the relevance of DNA testing in forensic casework.

### Impact of categorisation criteria

Shedder status category also varied significantly depending on the categorisation criteria used. As the number of possible shedder status designations increased from two to five, participants regularly moved between neighbouring categories, suggesting that an increased number of classification categories may better describe the shedding ability of participants. Most commonly, participants moved between two adjacent shedder classes, but participants shifted between three classes on two instances when five shedder categories were used (i.e. from low to low-intermediate to intermediate) and on two additional instances when three categories (i.e. from low to intermediate to high) were used. Therefore, a single high shedder replicate does not exclude the possibility of an overall low shedder designation and vice-versa.

When considering the use of categorisation criteria, thought must be given to the test parameters used. Within Test 1, where categorisation criteria were overlapping rather than discrete, most participants fell into said overlap for one or more test parameters. Being that participants had to reach a certain threshold in at least two of DNA, alleles, or RFU generated, ambiguity often arose, increasing the likelihood of misclassification. Additionally, variations in deposits may be drowned out or amplified by modest changes in behaviour or testing environment. The parameter ranges outlined in Table 1 were also reflective of the participants assessed in Goray et al. [15]. As more shedder data becomes available, parameter boundaries and the subsequent overlap may shift accordingly.

While Goray et al. [15] considered DNA, allelic, and RFU content to be primary, weight-equivalent parameters and mixture proportions to be a secondary parameter, Fonneløp et al. [18] only analysed DNA and allelic content when assigning shedder statuses. As no systematic review has been completed, there is currently no agreement on which parameters are most relevant to shedder status designation [47].

To be accurate, discrete shedder types need to reflect the proportion of low, intermediate, and high shedders seen in the general population. As highlighted in Table 6, when fingermarks were assessed using Fonneløp et al.’s modified cell counting method [18], which compared an individual’s average deposit to the distribution mean of all participants rather than pre-determined thresholds, a decrease in shedder status was seen for all participants in one, if not both, conditions. Further, the inconsistencies in Table 7 speak to the importance of standardising shedder criteria. Where Kanokwongnuwut et al. [11] may have over-estimated an individual’s shedder status when three frames were assessed, it seemingly underestimates one’s status when the entire fingermark is assessed at 50 × magnification, as all participants were low shedders. Comparatively, as modified Fonneløp et al. [18] and the in-house sample-adjusted method utilised shedder proportions specific to the sample being assessed, these methods generated a larger distribution of intermediate and high shedders.

Ultimately, the number of shedder designations used should have a practical implication on forensic casework, assisting forensic biologists with activity level evaluations and probability assessments regarding what profile types (i.e. quantity, quality, mixture proportions, presence of foreign DNA, etc.) may be found at a crime scene or on an item of evidence after contact by a particular shedder. If discrete categories are to be used by future forensic researchers, the number of categories used should sufficiently address these concerns. However, Petcharoen et al. [32] recently collated Diamond™ Dye data from 100 participants to show that participants often span multiple shedder categories and that the overlapping data is best represented by a continuum rather than discrete categories with arbitrary boundaries. Although, no large-scale DNA-based population studies have been conducted, Goray et al. [17] has previously suggested the existence of a similar continuum. Moving forward, data generated from a standardisation of shedder testing and categorisation will still better define a shedder continuum.

### Use of replicates

As highlighted in Table 8, participants often fluctuated in shedder status between replicates, a trend described in multiple previous studies [21, 27, 29, 31]. Being that only three replicates were used, no measurements could be reliably excluded as outliers; however, this variability suggests that three replicants is insufficient to determine an individual’s shedder status. Jansson et al. has shown that three measurements generate the same overall status designation as forty measurements approximately 60% of the time for a low shedder, 70% for an intermediate shedder, and 80% or more for a high shedder [48]. When the number of replicates increased to 12, the probability of being assigned to the correct shedder class was above 97% for five of their six participants [48]. Similar analyses of this type are desirable for each of the three methodologies applied in this study, as well as any other eventual standardised methodology used, to determine the predictive accuracy per number of tests conducted given an individual’s relative position within the shedder continuum. Although we recognise the infeasibility of requiring 12 sample deposits per person of interest in forensic casework, administering a minimum number of shedder tests may be reasonably conducted during criminal proceedings, along with buccal swabs or fingerprinting.

## Conclusion

Although shedder status data has been available and widely commented on for two decades, it appears far from being implemented effectively within the criminal justice system. This study highlights how shedder test selection impacts participant results, as none of our six participants maintained their shedder status between three different test types. Even within the same test type, as the number of possible status designations increased from two to five, most participates fluctuated in status between replicates. Therefore, the minimum number of replicates needed for accurate classification of shedders merits further evaluation. This study also highlights the impact of shedder categorisation criteria, as changing thresholds may be as relevant as DNA or cellular contribution to a shedder result; however, categorisation criteria is often overlooked and modified between studies without explanation or critique.

Moving forward, instead of finding new ways to source shedder data, there needs to be greater focus on ways to compare it. Ideally, forensic scientists would select and encourage the use of a single shedder test and set of categorisation criteria, as only a large-scale testing regime will generate the much-needed population data. If a shedder continuum is to be used instead of discrete categorisation criteria, relevant and comparable population data remains desperately needed to inform such a distribution.

## Supplementary Information

Below is the link to the electronic supplementary material.

Supplementary  File 1 (PDF 189 KB)

Supplementary File 2 (PDF 135 KB)

Supplementary File 3 (PDF 191 KB)

Supplementary File 4 (PDF 143 KB)

Supplementary File 5 (PDF 147 KB)

## Data Availability

The authors confirm that the data supporting the findings of this study are available within the article and its supplementary materials.
