# Supplementary material for: How to best assess shedder status: a comparison of popular shedder tests
Source: Int J Legal Med. 2024 Nov 7;139(3):965–81. doi: 10.1007/s00414-024-03351-8 (PMC12003581; doi:10.1007/s00414-024-03351-8)
Supplement: Supplementary file 1 — (PDF 189 KB) [file 414_2024_3351_MOESM1_ESM.pdf]

HOW TO BEST ASSESS SHEDDER STATUS: A COMPARISON OF POPULAR SHEDDER TESTS

Darya Ali[a*], Roland A.H. van Oorschot[b,c], Adrian Linacre[d], Mariya Goray[d]

[a] College of Medicine and Public Health, Flinders University, Bedford Park, South Australia, Australia
[b] Office of the Chief Forensic Scientist, Victoria Police Forensic Services Department, Macleod, Victoria, Australia
[c] School of Agriculture, Biomedicine and Environment, La Trobe University, Bundoora, Victoria, Australia
[d] College of Science and Engineering, Flinders University, Bedford Park, South Australia, Australia

***Corresponding Author:** Darya Ali, College of Medicine and Public Health, Flinders Medical Centre, Flinders Drive, Bedford Park South Australia 5042, GPO Box 2100 Adelaide SA 5000. Email: ali0242@flinders.edu.au

Supplementary Data 1: List of peer-reviewed shedder tests described in the literature.

Author	Shedder Test	Hand Used	Restrictions Prior to Sampling	Monitored Transfer Factors	Participant # Used	Replicate # Used	Classification Criteria	Classification Scheme	Classification Outcome
Lowe et al. (2002)	Hold 50 mL plastic tube (10 sec)	Not specified	15 mins post handwashing	Sex, handwashing	30	5	Median full donor profile=good	Binary (good/bad)	18 good, 12 bad shedders
Alessandrini et al. (2003)	Finger mark on glass, wood, metal surface (30 sec)	Not specified	Immediately post handwashing with soap or without handwashing	Nil specified	11	3	1. Complete profiles and high DNA yields=good<br>2. Partial or no DNA profiles=bad	Binary (good/bad)	3 good, 8 bad shedders
Phipps and Petricevic (2007)	Hold 50 mL plastic tube (10 sec)	Both hands	15 mins post handwashing with soap, hands dried with paper towel	Sex, handedness, oral intake, glove use, touching others	60	1	According to Lowe et al. for both hands.	Binary (good/bad)	0 good, 60 bad shedders
		Non-dominant					According to Lowe et al. for one hand.		8 good, 52 bad shedders
Allen et al. (2008)	Thumbprint on a glass slide (5 sec)	Both hands	Minimum 2 hours post handwashing	Sex	129	3	Based upon relative proportion of a DNA profile generated from fingerprint:<br>1. $\geq$300 pg DNA yield=heavy<br>2. 50-300 pg DNA =intermediate<br>3. $\leq$50 pg DNA=light	Ternary (heavy/ intermediate/ light)	24 heavy, 78 intermediate shedders, 27 light shedders
Djuric et al. (2008)	Hold plastic tube (10 sec)	Dominant	15 mins post handwashing	Nil specified	7	1	According to Lowe et al.	Binary (good/bad)	3 good, 4 bad shedders
Farmen et al. (2008)	Direct swab of palm	Both hands	No handwashing in AM prior	Nil specified	9	2	Modified Lowe et al., not otherwise defined.	Ternary (good/ medium/poor)	Not well defined - 7 with "good

Supplementary Data 1: List of peer-reviewed shedder tests described in the literature.

									analysable results," 2 poor shedders.
Graham and Rutty (2008)	Hold 25 mL plastic tube (30 sec)	Dominant	15 mins post handwashing	Sex, handedness, glove use	24	3	According to Lowe et al.	Binary (good/bad)	5 good, 19 bad shedders
Daly et. al (2012)	Grip glass, fabric or wood in fist (1 min), tape-lifting of material	No preference	No restrictions	Sex	100/ material type	1	According to Lowe et al.	Binary (good/bad)	39 good, 61 bad shedders
							Useful DNA profile of ≥6 alleles per deposition=good		66 good, 33 bad shedders
Quinones and Daniel (2012)	Rub hands together with 10g of glass beads, hand scrapings collected	Both hands involved	Handwashing with soap then wore vinyl gloves for 15 mins	Sex, age, race	8	1	Nil defined	Nil defined due to limited information regarding temperature, diet, physical state	Nil defined
Oleiwi et al. (2015)	Two finger (middle, ring) deposit on a glass plate (15 sec)	Not defined	1 hour post handwashing with soap, participants remained inactive during the wait period	Deposition pressure (kept at 4900 Pa)	6	4	Nil defined	Nil defined	Nil defined<br><br>Results suggest that one participant outperformed other five.
Goray et al. (2016)	Handprint on a glass plate (10 sec)	Both hands	No restrictions	Sex, age, handedness, handwashing, sneezing, nail-biting, moisturizer use,	10	3/day x 4 days	Based on the total amount of DNA (ng) deposited, alleles deposited, and mixture proportions.	Ternary (good/ intermediate/bad)	2 good, 6 intermediate, 2 bad shedders

Supplementary Data 1: List of peer-reviewed shedder tests described in the literature.

				recent sexual activity					
Manoli et al. (2016)	Hold 50 mL plastic tube (5 mins)	Each hand tested	No handwashing or glove use for 2-3 hours prior to testing	Sex, age, handedness	128	3 + 3	1. >80% donor profile=good<br>2. ≥ 41-80% =intermediate<br>3. ≤40%=bad	Ternary (good/ intermediate/ bad)	31 good, 64 intermediate, 33 bad shedders
Fonneløp et al. (2017)	Hold 15 mL plastic tube (10 sec)	Dominant	No restrictions	Sex, age, handedness, handwashing, glove use	20	3	2 out of 3 replicates DNA quantity ≥ mean + profile quality high (12 or more full loci) =good and all others bad.	Binary (good/bad)	5 good, 15 bad shedders
Kanokwongnuwut et al. (2018)	Thumbprint on a glass slide (15 sec)	Both thumbs	0, 15, 60, 180 mins post-handwashing	Sex	11	3	As shown in Figure 2:<br>1. >30 cells deposited with complete DNA profiles generated=heavy<br>2. 16-30 cells deposited with full or partial DNA profiles generated =intermediate<br>3. ≤15 cells deposited with partial DNA profiles generated =light	Ternary (heavy/ intermediate/ light)	2 heavy, 5 intermediate, 4 light shedders
Otten et al. (2019)	Hold 15 mL plastic tube (10 sec)	Dominant	No restrictions	Sex, age, handedness, handwashing	40	3	According to Fonneløp et al.	Binary (good/bad), 3 participants excluded from classification	12 good, 25 bad shedders, 3 couldn't be defined due to inconsistencies in test result.

Supplementary Data 1: List of peer-reviewed shedder tests described in the literature.

Rolo et al. (2019)	Hold plastic tube (30 sec)	Right	No handwashing or glove use for 1 hour prior	Nil specified	10	3	Regardless of contact area and substrate:<br>1. Complete profiles in ≥7/9 samples =good<br>2. Complete profiles in ≤2/9 samples =bad<br>3. Remaining =intermediate	Ternary (good/ intermediate/ poor)	1 good, 7 intermediate, 2 poor shedders
	Thumbprint on a glass plate (30 sec)					3			
	Handprint on a glass plate (30 sec)					3			
Tan et al. (2019)	Hold 50 mL plastic tube (10 sec)	Both hands	15 mins post handwashing with soap, hands dried with paper towel	Sex, age, handedness, oral intake, glove use, physical contact with others	81	2/day x 3 days	1. ≥ 4 out of 6 replicates with ≥ 16 detected alleles (reportable profile) =good<br>2. 1–3 reportable profiles =intermediate<br>3. 0 reportable profiles=bad	Ternary (good/ intermediate/bad)	~9 good (11%), 33 (41%) intermediate, 39 (49%) bad shedders
Burrill, Daniel and Frascione (2020)	Hands washed with PBS, aliquots collected	Both hands involved	Washed hands with soap, air dry	Nil specified	6	3	Nil defined	Nil defined	Nil defined
			Unwashed hands		6	3			
Kanokwongnuwut, Kirkbride & Linacre (2020)	Thumbprint on a glass slide (15 sec)	Not specified	0, 2, 15, 60 mins post-handwashing, dried with paper towel	Sex	3	3	According to Kanokwongnuwut et al. (2018)	Ternary (heavy/ intermediate/ light)	1 heavy, 1 intermediate, 1 light shedder
Goray and van Oorschot (2021)	Handprint on a glass	Both hands	No restrictions	Sex, age, handedness, handwashing,	10	3/day x 4 days	According to Goray et al. (2016).	Quinary (high/ intermediate-low /intermediate/	1 high, 2 intermediate-high, 3

Supplementary Data 1: List of peer-reviewed shedder tests described in the literature.

	plate (10 sec)			sneezing into hands, nail-biting, moisturizer use, recent sexual activity			No specific criteria defined for quinary classification.	intermediate-low/low)	intermediate, 2 intermediate-low, 2 low shedders
Goray and van Oorschot (2021)	Direct swab of palm	Both hands	30 mins post-handwashing with soap		4	2	Complete donor profiles=good	Binary (good/bad)	4 good, 0 bad shedders
Johannessen et al. (2021)	Hold 15 mL plastic tube (10 sec)	Dominant	Minimum one-hour post-handwashing		20	3	1. Above average total RFU for all participants in ≥2/3 replicates and ≥40 detected alleles in each profile=high<br>2. RFU of ≤10,000 and partial profiles in all samples=low<br>3. Remaining =medium	Ternary (high/ medium/low)	5 high, 10 medium, 5 low shedders
	Finger mark on a glass slide (3-5 sec)	Dominant	Minimum one-hour post-handwashing		20	2	1. ≥75% percentile of all cell deposits made by all participants=high<br>2. ≤25% percentile=low<br>3. Remaining =medium	Ternary (high/ medium/low)	5 high, 10 medium, 5 low shedders
Schmidt et al. (2021)	Hold 15 mL plastic tube (10 sec)	Dominant	No restrictions	Sex, age, handedness, skin conditions, handwashing, moisturizer use, glove use	15	3	According to Fonneløp et al.	Binary (good/bad)	5 good, 15 bad shedders

Supplementary Data 1: List of peer-reviewed shedder tests described in the literature.

Kaesler, Kirkbridge, and Linacre (2022)	Thumbprint on a glass slide (15 sec)	No preference	15 mins post handwashing, hands dried with paper towel	Sex, handwashing, hand sanitizer use, glove use, oral intake	10	15+5	According to Kanokwongnuwut et al. (2018)	Ternary (heavy/ intermediate/ light)	3 heavy, 4 intermediate, 3 light shedders
Lee et al. (2023)	Gripped a 50 mL plastic tube (10 sec)	Each hand	15 mins post-handwashing with soap, dried with paper towel. Participants to wear medical-grade face mask during wait period.	Sex, glove use, touching others, oral intake objects contacted, time of day	81	2/day x 3 days	According to Tan et al. (2019).	Ternary (good/ intermediate/ poor)	~9 good (11.1%), 33 intermediate (40.7%), 39 poor shedders (48.1%)
Jansson et al. (2024)	Gripped a 50 mL plastic tube (30 sec)	Both hands	No restrictions	Seasonal change, sweat generated, time since last handwash and shower	6	20	According to Johannessen et al. (2021)	Ternary (high/ intermediate/ low)	3 high, 1 intermediate, 2 low shedders
