# Supplementary material for: How to best assess shedder status: a comparison of popular shedder tests
Source: Int J Legal Med. 2024 Nov 7;139(3):965–81. doi: 10.1007/s00414-024-03351-8 (PMC12003581; doi:10.1007/s00414-024-03351-8)
Supplement: Supplementary file 2 — (PDF 135 KB) [file 414_2024_3351_MOESM2_ESM.pdf]

HOW TO BEST ASSESS SHEDDER STATUS: A COMPARISON OF POPULAR SHEDDER TESTS

Darya Ali[a*], Roland A.H. van Oorschot[b,c], Adrian Linacre[d], Mariya Goray[d]

[a] College of Medicine and Public Health, Flinders University, Bedford Park, South Australia, Australia
[b] Office of the Chief Forensic Scientist, Victoria Police Forensic Services Department, Macleod, Victoria, Australia
[c] School of Agriculture, Biomedicine and Environment, La Trobe University, Bundoora, Victoria, Australia
[d] College of Science and Engineering, Flinders University, Bedford Park, South Australia, Australia

***Corresponding Author:** Darya Ali, College of Medicine and Public Health, Flinders Medical Centre, Flinders Drive, Bedford Park South Australia 5042, GPO Box 2100 Adelaide SA 5000. Email: ali0242@flinders.edu.au

Supplementary Data 2: Characteristics of shedder participants.

Participant #	Gender:	Age Range:	Handedness:	Living Arrangements:	Skin Conditions:	Nail-biting?	Moisturizer Use	Gloves used throughout workday?	Previous Shedder Status Categorisation:
1	Female	36-45	Left	Alone	Nil	No	Daily - evenings	Yes	Unknown
2	Male	56-65	Left	Alone	Nil	No	Never	Yes	High
3	Female	18-25	Right	Alone	Transient rash on non-dominant hand *(potentially affecting 3 deposits)*	No	Never	Yes	Low
4	Female	18-25	Right	With roommates, children	Nil	No	Daily - evenings	Yes	Unknown
5	Female	18-25	Right	With partner, roommates	?Eczema – undiagnosed<br><br>Wound on fingers *(potentially affecting 1 deposit)*	Yes	Daily - mornings	Yes	Unknown
6	Male	18-25	Left	With parents, sibling	Nil	No	Never	Yes	Unknown
