# Supplementary material for: How to best assess shedder status: a comparison of popular shedder tests
Source: Int J Legal Med. 2024 Nov 7;139(3):965–81. doi: 10.1007/s00414-024-03351-8 (PMC12003581; doi:10.1007/s00414-024-03351-8)
Supplement: Supplementary file 3 — (PDF 191 KB) [file 414_2024_3351_MOESM3_ESM.pdf]

HOW TO BEST ASSESS SHEDDER STATUS: A COMPARISON OF POPULAR SHEDDER TESTS

Darya Ali[a*], Roland A.H. van Oorschot[b,c], Adrian Linacre[d], Mariya Goray[d]

[a] College of Medicine and Public Health, Flinders University, Bedford Park, South Australia, Australia
[b] Office of the Chief Forensic Scientist, Victoria Police Forensic Services Department, Macleod, Victoria, Australia
[c] School of Agriculture, Biomedicine and Environment, La Trobe University, Bundoora, Victoria, Australia
[d] College of Science and Engineering, Flinders University, Bedford Park, South Australia, Australia

***Corresponding Author:** Darya Ali, College of Medicine and Public Health, Flinders Medical Centre, Flinders Drive, Bedford Park South Australia 5042, GPO Box 2100 Adelaide SA 5000. Email: ali0242@flinders.edu.au

Supplementary Data 3: A detailed review of the methodology used in the three original studies being replicated and any modifications made in Tests 1-3.

Shedder Test	Participant #	Replicant #	Cleaning Protocols and Negative Controls	Pre-deposit Restrictions	Methodology	Sampling and Downstream Processing	Result Interpretation
**Goray and van Oorschot (2021)**	10	3 replicates per day (morning, mid-morning, afternoon) x 4 days	Glass plates cleaned with 1% hypochlorite and distilled water.<br><br>Control swabs taken from all plates and random swabs quantified.	No restrictions.	Participants asked to deposit both left and right handprints onto cleaned glass plates (dimensions: 140 × 220 mm; 4 mm thickness), with fingers relatively close together, with some pressure but no friction for 10 seconds.	Plates swabbed with a wet and dry double swab technique (150C, Copan©).<br><br>DNA extracted with DNA IQ™ system, quantified using the Quantifiler Trio™ kit, and amplified using PowerPlex® 21.<br><br>PCR products run on a 3500xL Genetic Analyser and analysed using GeneMapper™ IDx Software.	Total DNA recovered separated into donor and non-donor DNA from the percent contributions as determined by STRmix™. Mixture proportions calculated and used to assign major and minor contributors, with major contributor defined as an individual depositing over 70% of the total DNA detected in the mixed sample. Contributions to mixed profiles where no major was assigned were considered as unresolved mixtures.<br><br>Shedder status based on the total amount of DNA (ng) deposited, alleles deposited, and mixture proportions generated. No specific criteria defined for quinary classification.
**Test 1 – Handprint on a Glass Plate**	6	3 (dirty) + 3 (clean), each taken on different days	Glass plates cleaned with 1% hypochlorite, 100% ethanol, and distilled water.<br><br>Control swabs taken from random plates and quantified.	Participants washed their hands for 15-20 seconds with soap and dried with paper towel. Deposits made 15 minutes post-handwashing.<br><br>During wait-period, participants either used their hands *(dirty condition)* but were to refrain from re-washing, sanitizing, eating and drinking, or actively touching others, or were observed and kept from using their hands *(clean condition).*	Participants asked to deposit their dominant handprint on a cleaned glass plate (dimensions: 140 × 220 mm; 4 mm thickness), with fingers relatively close together, with some pressure but no friction for 10 seconds.	Plates swabbed with a wet and dry double swab technique (155C, Copan©).<br><br>DNA was extracted with the DNA IQ™ system, quantified using the Quantifiler® Trio DNA Quantification Kit on an ABIPRISM® 7500 and amplified using PowerPlex® 21.<br><br>PCR products run on a 3500 Genetic Analyser and analysed using GeneMapper® ID-X software (v1.6).	Mixture proportions determined identically to Goray and van Oorschot (2021).<br><br>Shedder status based on total amount of DNA (ng) and alleles deposited, RFU, and mixture proportions generated. Quinary classification assigned as per Table 1 (as modified from Goray and van Oorschot, 2021).

Supplementary Data 3: A detailed review of the methodology used in the three original studies being replicated and any modifications made in Tests 1-3.

Shedder Test	Participant #	Replicant #	Cleaning Protocols and Negative Controls	Pre-deposit Restrictions	Methodology	Sampling and Downstream Processing	Result Interpretation
**Fonneløp et al. (2017)**	20	3, each taken on different days	Equipment cleaned with 0.1% hypochlorite or RNase AWAY™.	No restrictions.<br><br>Participants asked to recall time since last handwash and glove use.	Participants were asked to hold a plastic conical tube (VWR ®,15 mL) for 10 seconds using their dominant hand.	Plastic tubes transferred to a clean bag until sampling. Body of each plastic tube was swabbed with a wet swab (brand not specified), while the caps remained unsampled<br><br>DNA extracted using a 5% Chelex® procedure, quantified using the Quantifiler® Trio Kit on the 7500 Real-Time PCR system, and amplified using PowerPlex® ESX 17 Fast System.<br><br>PCR products run on a 3500xl Genetic Analyser and analysed using GeneMapper® ID-X software.	DNA analysis, including number of contributors, not further described.<br><br>Shedder status based on the following criteria: good shedders deposited DNA quantities that were greater than the mean for all participants and high DNA qualities (defined as 12+ full loci) in 2 or more replicates. All other participants were low shedders.
**Test 2 – Grip Mark on a Plastic Tube**	6	3 (dirty) + 3 (clean), each taken on different days	Plastic tubes cleaned with 1% hypochlorite, 100% ethanol, and distilled water.<br><br>Control swabs taken from random tubes and quantified.	Participants washed their hands for 15-20 seconds with soap and dried with paper towel. Deposits made 15 minutes post-handwashing.<br><br>During wait-period, participants either used their hands *(dirty condition)* but were to refrain from re-washing, sanitizing, eating and drinking, or actively touching others, or were observed and kept from using their hands *(clean condition).*	Participants were asked to grip a cleaned plastic conical tube (Greiner centrifuge tube, 50 mL) with firm pressure for 10 seconds using their dominant hand.<br><br>Participants asked to ensure that their entire hand spanned the body of the tube and that no part touched the cap.	Body of each plastic tube was immediately swabbed with a wet and dry double swab technique (155C, Copan©), while the caps remained unsampled.<br><br>DNA was extracted with the DNA IQ system, quantified using the Quantifiler® Trio DNA Quantification Kit on an ABIPRISM® 7500 and amplified using PowerPlex® 21.<br><br>PCR products run on a 3500 Genetic Analyser and analysed using GeneMapper® ID-X software (v1.6).	Total DNA recovered separated into donor and non-donor DNA from the percent contributions as determined by STRmix™.<br><br>Shedder status based on DNA quantity and profile quality as per Fonnelop et al. (2017). To account for the use of PowerPlex 21, 15 or more full loci needed to meet the threshold for a “high quality” profile.

Supplementary Data 3: A detailed review of the methodology used in the three original studies being replicated and any modifications made in Tests 1-3.

Shedder Test	Participant #	Replicant #	Cleaning Protocols and Negative Controls	Pre-deposit Restrictions	Methodology	Sampling and Downstream Processing	Result Interpretation
**Kanokwong-nuwut et al. (2018)**	11	3, each taken on different days	Glass slides cleaned with 3% bleach, ethanol, and irradiated with UV light for 15 minutes.<br><br>Random negative control slides stained with DD.	Participants washed their hands (no soap) and dried with paper towel. Deposits made 15 minutes post-handwashing.<br><br>No restrictions during wait period. Participants instructed to carry on with normal daily activities.	Participants placed both thumbs on cleaned DNA-free glass slides with medium pressure for 15 seconds.	5 µL of DD (20-fold dilution of DD stock) pipetted onto glass slide and spread with pipette tip.<br><br>Once dried, cells were visualised using a Dino-Lite fluorescent digital microscope under blue light (480 nm) on a black background at 220x magnification.<br><br>Cellular material in three 1mm^2 squares of high density were visualised using DinoXcope 2 for Mac OS. The selected squares were then scored.	Participants who deposited between 1-15 cells/mm^2 were considered light shedders, those who deposited between 16-30 cells/mm^2 were intermediate shedders, and those who deposited over 30 cells/mm^2 were high shedders.
**Test 3 – Fingermark on a Glass Slide**	6	3 (dirty) + 3 (clean), each taken on different days	Glass slides cleaned with 100% ethanol.<br><br>Random negative control slides stained with DD.	Participants washed their hands for 15-20 seconds with soap and dried with paper towel. Deposits made 15 minutes post-handwashing.<br><br>During wait-period, participants either used their hands *(dirty condition)* but were to refrain from re-washing, sanitizing, eating and drinking, or actively touching others, or were observed and kept from using their hands *(clean condition).*	Conducted directly after Test 2.<br><br>Participants placed their dominant thumb on a cleaned DNA-free glass slide with medium pressure for 15 seconds.	10 µL of DD (20-fold dilution of the DD stock) pipetted onto glass slide and spread with pipette tip.<br><br>After being left to dry for 30 minutes, cells were visualised using Dino-Lite fluorescent digital microscope under blue light (480 nm) on a black background at 220x magnification.<br><br>Cellular material scored as per Kanokwongnuwut et al. (2018).	As per Kanokwongnuwut et al. (2018).
