# Supplementary material for: How to best assess shedder status: a comparison of popular shedder tests
Source: Int J Legal Med. 2024 Nov 7;139(3):965–81. doi: 10.1007/s00414-024-03351-8 (PMC12003581; doi:10.1007/s00414-024-03351-8)
Supplement: Supplementary file 4 — (PDF 143 KB) [file 414_2024_3351_MOESM4_ESM.pdf]

HOW TO BEST ASSESS SHEDDER STATUS: A COMPARISON OF POPULAR SHEDDER TESTS

Darya Ali[a*], Roland A.H. van Oorschot[b,c], Adrian Linacre[d], Mariya Goray[d]

[a] College of Medicine and Public Health, Flinders University, Bedford Park, South Australia, Australia
[b] Office of the Chief Forensic Scientist, Victoria Police Forensic Services Department, Macleod, Victoria, Australia
[c] School of Agriculture, Biomedicine and Environment, La Trobe University, Bundoora, Victoria, Australia
[d] College of Science and Engineering, Flinders University, Bedford Park, South Australia, Australia

***Corresponding Author:** Darya Ali, College of Medicine and Public Health, Flinders Medical Centre, Flinders Drive, Bedford Park South Australia 5042, GPO Box 2100 Adelaide SA 5000. Email: ali0242@flinders.edu.au

<u>Supplementary Data 4:</u> Activities conducted by all participants (across all tests and replicates) during the 15-minute wait period in the dirty hands condition, as self-reported by participants on the day of testing.

	**Handprint on a Glass Plate (Test 1)**					
	**Replicate 1**		**Replicate 2**		**Replicate 3**	
**Participant**	**Spaces Occupied**	**Activities Conducted**	**Spaces Occupied**	**Activities Conducted**	**Spaces Occupied**	**Activities Conducted**
1	Hallway, personal office	Office and computer work, touched face a couple of times	Hallway, personal office	Office and computer work, touched face a couple of times	Hallway, personal office	Office and computer work, touched face a couple of times
2	Hallway, shared student office	Spoke to two students	Hallway, outdoor courtyard, personal office	Grading papers	Hallway, outdoor courtyard, personal office	Grading papers
3	Hallway, shared office (with 1 other)	Office work, typing on a computer, messaging on a smartphone, scratched neck once	Hallway, shared office (with 2 others)	Office work, touched face 2 times	Hallway, shared office (with 1 other)	Office work, typing on personal and shared laptop, touched face 3 times
4	Hallway, shared office (with 2 others)	Talking, touched face twice	Hallway, shared office	Talking, touched face twice	Hallway, shared office	Wrote in lab book, touched face once
5	Hallway, shared office (with 3 others)	Writing on tablet, messaging on smart phone	Hallway, shared office	Messaging on smart phone	Hallway, shared office (with 1 other)	Computer work with 1 other where both where touching keyboard/mouse, touched face a few times
6	Hallway, shared office	Unlocked office door, computer work on personal laptop, messaging on smart phone	Hallway, shared office	Computer work on personal laptop, took a drink out of water bottle, touched face 2 times	Hallway, shared office	Unlocked office door, computer work on personal laptop

Supplementary Data 4: Activities conducted by all participants (across all tests and replicates) during the 15-minute wait period in the dirty hands condition, as self-reported by participants on the day of testing.

	**Grip mark on a Plastic Conical Tube (Test 2) and Fingermark on a Glass Slide (Test 3)**					
	**Replicate 1**		**Replicate 2**		**Replicate 3**	
**Participant**	**Spaces Occupied**	**Activities Conducted**	**Spaces Occupied**	**Activities Conducted**	**Spaces Occupied**	**Activities Conducted**
1	Hallway, personal office	Office work (using a recently cleaned keyboard), talking to a student	Hallway, personal office	Office work, talking to a colleague	Hallway, personal office	Office work, talking to a student, touched face a few times
2	Hallway, outdoor courtyard, personal office	Typing using a computer and mouse	Hallway, outdoor courtyard, personal office	Office work	Hallway, outdoor courtyard, personal office	Office work
3	Hallway, shared office (with 1 other)	Office work, typing on a computer, messaging on a smartphone, touched face once	Hallway, outdoor coffee cart (with 1 other)	Talking (with friend and barista), took a sip of coffee, touched face 3 times	Hallway, shared office (with 1 other)	Office work, typing on personal and shared laptop, touched face 2 times
4	Hallway, shared office	Talking	Hallway, shared office	Talking, touched face once	Hallway, shared office	Pacing around office
5	Hallway, shared office (with 3 others)	Writing on tablet, messaging on smart phone, touched face a couple of times	Hallway, shared office (with 2 others)	Writing on tablet, messaging on smart phone	Hallway, shared office (with 2 others)	Writing on tablet, messaging on smart phone
6	Hallway, shared office	Computer work on personal laptop	Hallway, shared office	Computer work on personal laptop	Hallway, shared office	Unlocked office door, computer work on personal laptop
