# Supplementary material for: How to best assess shedder status: a comparison of popular shedder tests
Source: Int J Legal Med. 2024 Nov 7;139(3):965–81. doi: 10.1007/s00414-024-03351-8 (PMC12003581; doi:10.1007/s00414-024-03351-8)
Supplement: Supplementary file 5 — (PDF 147 KB) [file 414_2024_3351_MOESM5_ESM.pdf]

HOW TO BEST ASSESS SHEDDER STATUS: A COMPARISON OF POPULAR SHEDDER TESTS

Darya Ali[a*], Roland A.H. van Oorschot[b,c], Adrian Linacre[d], Mariya Goray[d]

[a] College of Medicine and Public Health, Flinders University, Bedford Park, South Australia, Australia
[b] Office of the Chief Forensic Scientist, Victoria Police Forensic Services Department, Macleod, Victoria, Australia
[c] School of Agriculture, Biomedicine and Environment, La Trobe University, Bundoora, Victoria, Australia
[d] College of Science and Engineering, Flinders University, Bedford Park, South Australia, Australia

***Corresponding Author:** Darya Ali, College of Medicine and Public Health, Flinders Medical Centre, Flinders Drive, Bedford Park South Australia 5042, GPO Box 2100 Adelaide SA 5000. Email: ali0242@flinders.edu.au

<u>Supplementary Data 5:</u> Test 2 replicate results for all participants, as adapted from Fonneløp et al. [18] using criteria from Goray & van Oorschot [15], in the clean condition ("C") and dirty hands ("D") conditions. The number of contributors ("NOC") per replicate are denoted and all non-donors were considered "unknowns" for the purpose of analysis. Results that are unavailable ("N/A") reflect samples that did not generate a DNA deposit and/or profile. To be named the major donor, an individual must have contributed 70% or more of the total DNA generated within a mixture. The donor likelihood ratios are provided in the table.

		**NOC**		**Donor DNA (ng)**		**Donor Alleles (%)**		**Donor Average RFU**		**Mixture Proportions (Donor: Non-Donor)**		**Donor Major Contributor**		**Likelihood Ratio**	
**Participant**	**Replicate**	**C**	**D**	**C**	**D**	**C**	**D**	**C**	**D**	**C**	**D**	**C**	**D**	**C**	**D**
1	1	2	2	0.04	0.12	15	13	231	177	73:27	69:31	Yes	No	$3.5e^{2}$	$3.9e^{1}$
	2	N/A	1	0	0.06	0	7.5	0	229	N/A	100:0	N/A	Yes	N/A	$2.5e^{1}$
	3	1	1	0	0.24	3	30	179	409	100:0	100:0	Yes	Yes	$2.3e^{0}$	$5.9e^{7}$
	**Average**	1	1	0.01	0.14	6	17	137	272						
2	1	2	1	0.35	0.240	80	48	631	422	83:17	100:0	Yes	Yes	$6.7e^{18}$	$1.3e^{13}$
	2	1	2	0.06	2.43	15	95	331	2581	100:0	99:1	Yes	Yes	$4.6e^{3}$	$5.6e^{24}$
	3	1	2	3.00	1.77	100	98	361	2402	100:0	98:2	Yes	Yes	$4.6e^{26}$	$2.6e^{26}$
	**Average**	1	2	1.14	1.48	65	80	441	1802						
3	1	2	2	0.08	0.13	48	53	217	292	67:33	70:30	No	Yes	$5.0e^{8}$	$2.5e^{9}$
	2	2	2	0.04	0.22	5	50	160	423	72:28	73:27	Yes	Yes	$10e^{0}$	$1.4e^{15}$
	3	1	1	0.06	0.12	5	28	197	282	100:0	100:0	Yes	Yes	$10e^{0}$	$4.5e^{8}$
	**Average**	2	2	0.06	0.16	19	43	191	332						
4	1	1	2	0.30	0.04	45	43	725	329	100:0	73:27	Yes	Yes	$7.8e^{11}$	$4.2e^{7}$
	2	2	1	0.17	0.60	25	50	401	483	73:37	100:0	Yes	Yes	$7.9e^{4}$	$5.3e^{11}$
	3	1	2	0.12	0.17	8	28	207	294	100:0	71:29	Yes	Yes	$5.0e^{1}$	$1.2e^{4}$
	**Average**	1	2	0.20	0.27	26	40	444	369						
5	1	1	2	0	0.12	0	13	0	168	0:100	65:34	No	No	$4.6e^{-7}$	$7.6e^{0}$
	2	1	3	0.24	0.09	5	40	192	304	100:0	49:51	Yes	No	$1.5e^{1}$	$6.1e^{6}$
	3	N/A	2	0	1.07	0	100	0	1598	N/A	99:1	N/A	No	N/A	$6.5e^{26}$
	**Average**	1	2	0.08	0.43	2	51	64	690						
6	1	2	1	0.11	0.06	50	3	236	175	64:36	100:0	No	Yes	$1.7e^{10}$	$4.1e^{0}$
	2	N/A	1	0	0.12	0	43	0	423	N/A	100:0	N/A	Yes	N/A	$3.9e^{13}$

	3	1	1	0.06	0.30	18	53	378	573	100:0	100:0	Yes	Yes	$5.3e^{5}$	$6.8e^{15}$
	**Average**	1	1	0.06	0.16	23	33	205	390						
**Overall Participant Averages**		**1**	**2**	**0.26 ng**	**0.44 ng**	**23% alleles**	**44% alleles**	**247 RFU**	**642 RFU**						

.
